# Hyalocytes—guardians of the vitreoretinal interface

**DOI:** 10.1007/s00417-024-06448-3

**Published:** 2024-04-03

**Authors:** Clemens Lange, Stefaniya Boneva, Peter Wieghofer, J. Sebag

**Affiliations:** 1 Department of Ophthalmology, St. Franziskus Hospital, Muenster, Germany; 2 Eye Center, Medical Center, Faculty of Medicine, University of Freiburg, Freiburg, Germany; 3 Cellular Neuroanatomy, Institute of Theoretical Medicine, Medical Faculty, University of Augsburg, Augsburg, Germany; 4 VMR Institute for Vitreous Macula Retina, Huntington Beach, CA USA; 5 Doheny Eye Institute, UCLA, Pasadena, CA USA; 6 Department of Ophthalmology, Geffen School of Medicine, UCLA, Los Angeles, CA USA

**Keywords:** Hyalocytes, Vitreous, Immunology, Vitreoretinal interface

## Abstract

Originally discovered in the nineteenth century, hyalocytes are the resident macrophage cell population in the vitreous body. Despite this, a comprehensive understanding of their precise function and immunological significance has only recently emerged. In this article, we summarize recent in-depth investigations deciphering the critical role of hyalocytes in various aspects of vitreous physiology, such as the molecular biology and functions of hyalocytes during development, adult homeostasis, and disease. Hyalocytes are involved in fetal vitreous development, hyaloid vasculature regression, surveillance and metabolism of the vitreoretinal interface, synthesis and breakdown of vitreous components, and maintenance of vitreous transparency. While sharing certain resemblances with other myeloid cell populations such as retinal microglia, hyalocytes possess a distinct molecular signature and exhibit a gene expression profile tailored to the specific needs of their host tissue. In addition to inflammatory eye diseases such as uveitis, hyalocytes play important roles in conditions characterized by anomalous posterior vitreous detachment (PVD) and vitreoschisis. These can be hypercellular tractional vitreo-retinopathies, such as macular pucker, proliferative vitreo-retinopathy (PVR), and proliferative diabetic vitreo-retinopathy (PDVR), as well as paucicellular disorders such as vitreo-macular traction syndrome and macular holes. Notably, hyalocytes assume a significant role in the early pathophysiology of these disorders by promoting cell migration and proliferation, as well as subsequent membrane contraction, and vitreoretinal traction. Thus, early intervention targeting hyalocytes could potentially mitigate disease progression and prevent the development of proliferative vitreoretinal disorders altogether, by eliminating the involvement of vitreous and hyalocytes.


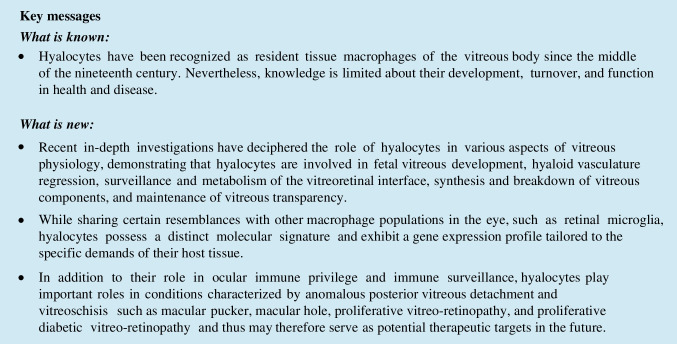


## Introduction

Hyalocytes are mononuclear phagocytes residing in the vitreous cortex that have fascinated researchers for nearly two centuries. Extensive scientific investigations have been conducted since the early 1800s to unravel the true essence and origins of hyalocytes, yielding diverse and subject-to-interpretation findings spawning numerous theories regarding their function. The initial identification of hyalocytes in 1840 is commonly attributed to Hannover, a Danish anatomist, who introduced hyalocytes as a distinct cell population of macrophages that resides within the vitreous body [1]. Rudolf Virchow observed these cells in 1852 and proposed that they are responsible for producing the extracellular matrix of the vitreous body [2]. In 1874, hyalocytes were identified as macrophages based on their morphology and the ability to proliferate after antigen inoculation [3, 4]. The hypothesis suggesting a developmental connection between hyalocytes and retinal microglia (known as Hortega cells at that time) was first proposed in 1931 [5]. However, the intricate relationship between hyalocytes, retinal microglia, and blood-derived monocytes remains enigmatic, spurring investigations utilizing diverse assays and models. Finally, the term “hyalocytes” was introduced by Balazs and colleagues to describe a homogeneous cell population in the cortical layer of the vitreous body throughout various animal species [6].

Despite the pioneering efforts of these early investigators, who were constrained by the limitations of then available methodologies, recent advances in imaging techniques [7, 8] and high-throughput transcriptional and protein analyses have provided a more comprehensive understanding of the unique characteristics of hyalocytes. The origin, turnover, localization, structure, and expression profile of hyalocytes have undergone examination using various techniques, including dark-field slit-, light-, phase contrast-, and electron microscopy; as well as immunohistochemistry, immunofluorescent labeling, transgenic reporter lines application combined with fluorescent microscopy, and confocal microscopy [9]. Furthermore, the utilization of proteomic studies including imaging mass cytometry has propelled our understanding forward [10–12]. While these techniques have produced invaluable insights into hyalocyte physiology, the advent of optical coherence tomography (OCT), scanning light ophthalmoscopy (SLO), and adaptive optics (AOSLO) has paved the way for imaging single hyalocytes in vivo over time, particularly in human subjects [13]. Collectively, these recent advances in available approaches have not only validated established knowledge but have also uncovered novel notions about the immunomodulation properties of this cell population. These findings demonstrate that although hyalocytes exhibit certain resemblances to other macrophages and microglial cells in specific tissue niches, they constitute a unique population specialized in meeting the specific needs of the vitreous body, most notably preserving transparency.

In this article, we summarize current understanding of the molecular biology and functions of hyalocytes during both development and adult homeostasis, including their roles in immune surveillance and privilege. Furthermore, we explore the role(s) of hyalocytes in various diseases and delve into the potentially beneficial and detrimental features of activated hyalocytes in proliferative vitreoretinal conditions. The scientific observations of the past decade raise intriguing questions regarding the dual nature of hyalocytes throughout an individual’s lifespan. What mechanisms govern the delicate balance between their advantageous and harmful properties? Do hyalocytes act as allies or adversaries in the pathophysiology of vitreoretinal disorders? Is it possible that multiple subpopulations of hyalocytes exist, with some being actively involved in disease while others remain quiescent until triggered by pathological conditions? These thought-provoking inquiries and others will be explored herein. However, it is important to note that definitive answers to these questions will likely necessitate further extensive scientific and clinical investigations during the years to come.

## Origin and turnover of hyalocytes

Starting from the 4th week of gestation in humans, mesodermal cells enter the developing eye through the ocular cleft and contribute to the formation of the hyaloid vascular system, which ultimately includes the hyaloid artery, the vasa hyaloidea propria, the tunica vasculosa lentis, and the pupillary membrane [14, 15]. Accompanying this vascular system, the mesodermal cells secrete extracellular components of the primary vitreous and express cell surface markers such as major histocompatibility complex (MHC) molecules and CD45, indicating their status as precursor cells to macrophages that eventually become hyalocytes [16]. After reaching its developmental peak around the 10th week of gestation, the hyaloid vasculature begins to regress [16]. This involution process, which is critical for creating a clear optical pathway, is mediated by primitive macrophages, i.e., hyalocyte precursor cells. Its failure can lead to significant impairments in vitreoretinal and ocular development. Experimental investigations indicate that dysfunction of these macrophages, such as through the use of toxic liposomes applied within the eye [17, 18] or through genetic manipulation, as observed in *PU.1*-deficient mice [19], is linked to a prolonged persistence of hyaloid vessels and the pupillary membrane, which normally represent transient structures. This evidence strongly suggests that macrophages and more specifically hyalocytes play a crucial role in the programmed remodeling of hyaloid vessels [14].

The intriguing phenomenon of hyalocyte regeneration within the confines of the blood-ocular barrier has sparked considerable curiosity, giving rise to a multitude of queries. The origin of hyalocytes and the mechanisms underlying their replenishment remain enigmatic. Does the pool of hyalocytes undergo a perpetually repeated cycle of apoptosis, followed by replenishment with mitotically active regenerating cells? Alternatively, could circulating progenitor cells, such as monocytes, infiltrate vitreous from the bloodstream and differentiate into hyalocytes, thus providing continued vibrance of this cell population? In recent decades, there has been a surge of research focusing on the origin and turnover of macrophages during both developmental stages and adulthood. This scientific exploration has been driven by the emergence of innovative techniques, including transferring labeled bone marrow cells after lethal irradiation to differentiate it from the recipient’s bone marrow, parabiosis, and fate mapping in transgenic mice [20, 21].

Early studies revealed that hyalocytes exhibit a low mitotic rate and do not incorporate ^3^H-thymidine when it is introduced into the eye. However, increased mitotic activity has been observed under pathologic conditions such as following retinal photocoagulation [22, 23]. Attempts have been made to replenish vitreous cells with hematogenous cells that transform into hyalocytes by transplanting labelled bone marrow cells from a transgenic mouse into a wild-type mouse. These endeavors achieved some success, implying that hyalocyte renewal predominantly occurs through the bloodstream [24]. Nevertheless, this technique has inherent limitations, as transplantation has only been feasible after lethal irradiation of the mice to deplete the recipients bone marrow before transplantation, potentially affecting the integrity of the blood-ocular barrier leading to side effects and an overestimation of the turnover rate. Additionally, intravenous transfer of bone marrow introduces exogenous hematopoietic stem cells and myeloid progenitors into the blood stream, resulting in promotion of artificial tissue infiltration and potentially confounding interpretations regarding the origin of these cells.

Another experimental approach involves parabiosis, a surgical technique that joins two mice to establish a shared circulation. This method enables leukocytes, including monocytes, to course between the two animals. Parabiosis offers advantages over bone marrow chimeras as it closely mimics the physiologic condition without the need for lethal irradiation. Utilizing parabiosis, investigations have suggested that hematogenous re-population of microglial cells in the retina and brain is improbable [25] suggesting that this may also be the case for hyalocytes.

Finally, conditional transgenic reporter mouse lines have been developed as valuable tools for investigating the turnover of resident macrophages by enabling the inducible removal of specific gene sequences. While some models have targeted microglia-specific genes, their application to hyalocytes remains unexplored. Identifying a distinct gene signature that is unique to hyalocytes, as suggested by Wolf and colleagues [26], would greatly enhance our understanding of their relationship to retinal microglia and other tissue macrophages.

In summary, diverse experimental approaches have significantly contributed to our understanding of macrophage turnover. Recent evidence indicates that hyalocytes possess long lifespans and that their regeneration, albeit at a slow pace, primarily occurs autonomously, similar to microglia [9]. However, further exploration utilizing these and other methods is needed to unravel the intricate dynamics of hyalocytes.

## Imaging hyalocytes

**Fig. 1:**
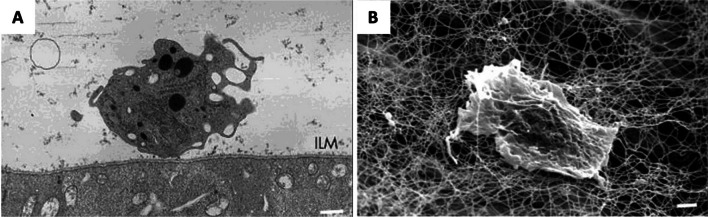
Human hyalocytes in situ and imaged by electron microscopy. **A** A hyalocyte within the posterior vitreous cortex, in proximity to the inner limiting membrane (ILM), as captured by transmission electron microscopy. The hyalocyte exhibits classic characteristics of cells of the macrophage lineage, e.g. lysosome-like granula, mitochondria, and micropinocytotic vesicles. **B** A scanning electron micrograph shows a hyalocyte with a few processes entangled in a collagen fiber network. Scale bars: **A** 6000 × , 1 µm, **B** 4300 × , 1 µm. . Reproduced with permission from Qiao et al., *Br J Ophthalmol* 2005 [24]

Initial investigations revealed that hyalocytes exhibit morphologic characteristics similar to macrophages when observed with electron microscopy [27, 28]. They possess a distinctive star-shaped structure with serrated edges, pseudopodia, a lobulated nucleus, a well-developed Golgi apparatus, and numerous lysosomal granules and phagosomes (Fig. 1). In the eye, hyalocytes are located within the vitreous cortex and align in parallel layers, forming a phalanx-like arrangement positioned at a variable distance anterior to the inner limiting membrane (ILM) and in proximity to the ciliary body. Hyalocytes can also be found floating freely within the vitreous body, although in smaller numbers.

**Fig. 2:**
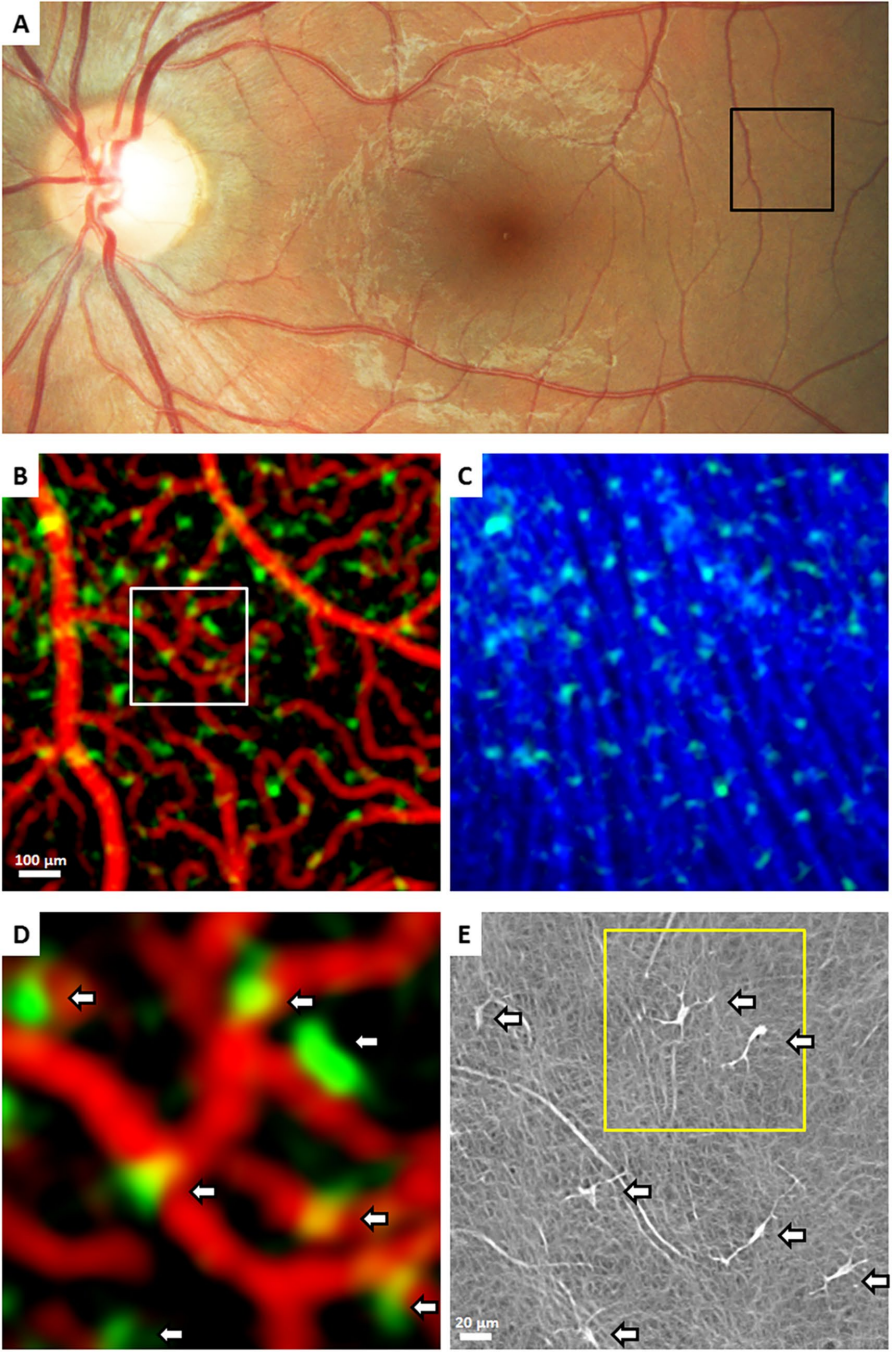
Human hyalocytes imaging in vivo. Clinical optical coherence tomography (OCT) and adaptive optics scanning light ophthalmoscopy (AOSLO) imaging of a 32-year-old male. **A** Color fundus photo. The black box indicates a region imaged using clinical OCT in **B**. **B**, **C** OCT reflectance and OCT angiography (OCTA) color overlays of the area within the black box in **A**. Spatial relationships between structures are visualized via clinical OCT color overlays of **B** the superficial retinal vascular network (red) and hyalocytes (green), and **C** hyalocytes (green) imaged anterior to the retinal nerve fiber bundles (blue). **D** Higher magnification of the color overlay of the superficial retinal vascular network (red) and hyalocytes (green) of the area within the white box in **B**. The white arrows indicate seven hyalocytes. **E** Corresponding AOSLO image reveals the same hyalocytes (white arrows), imaged with better visibility of their cell somas and processes. Hyalocyte locations match between imaging modalities, but the cell size and shape appear different. Reproduced with permission from Wieghofer et al., *Exp Rev Ophthalmol* 2022 [9]

Clinically, hyalocytes can be visualized using spectral-domain and swept-source OCT, appearing as highly reflective round dots, typically requiring minimal image enhancement. However, specific modifications to standard image acquisition and post-processing procedures can be advantageous for accurate localization of hyalocytes within the cortical vitreous fiber meshwork. Enhanced vitreous imaging (EVI) has been introduced to average a large number of scans using the automatic real-time function on the Heidelberg Spectralis device. Notably, coronal plane (en face) OCT imaging specifically visualizes hyalocytes in the cortical vitreous close to the retinal surface, while their presence is less prominent in more anterior regions of the vitreous body. Furthermore, recent advances in adaptive optics OCT imaging enable direct visualization of human hyalocytes in vivo (Fig. 2). With this innovative approach, researchers, such as Migacz et al. [8], described the dynamic movement and morphologic changes of hyalocyte cell bodies and processes within the living human eye. These studies demonstrate that hyalocytes have a branched morphology with projections resembling filopodia that exhibit continuous movement suggestive of a role in environmental monitoring. The motion of hyalocytes themselves occurs in rapid bursts, averaging a speed of 0.23 ± 0.29 µm/min and reaching a maximum of 2.0 µm/min. It appears that hyalocytes operate within a relatively confined area, with their projections constantly extending and retracting as they survey their surroundings for foreign antigens.

## Hyalocyte functions

**Fig. 3:**
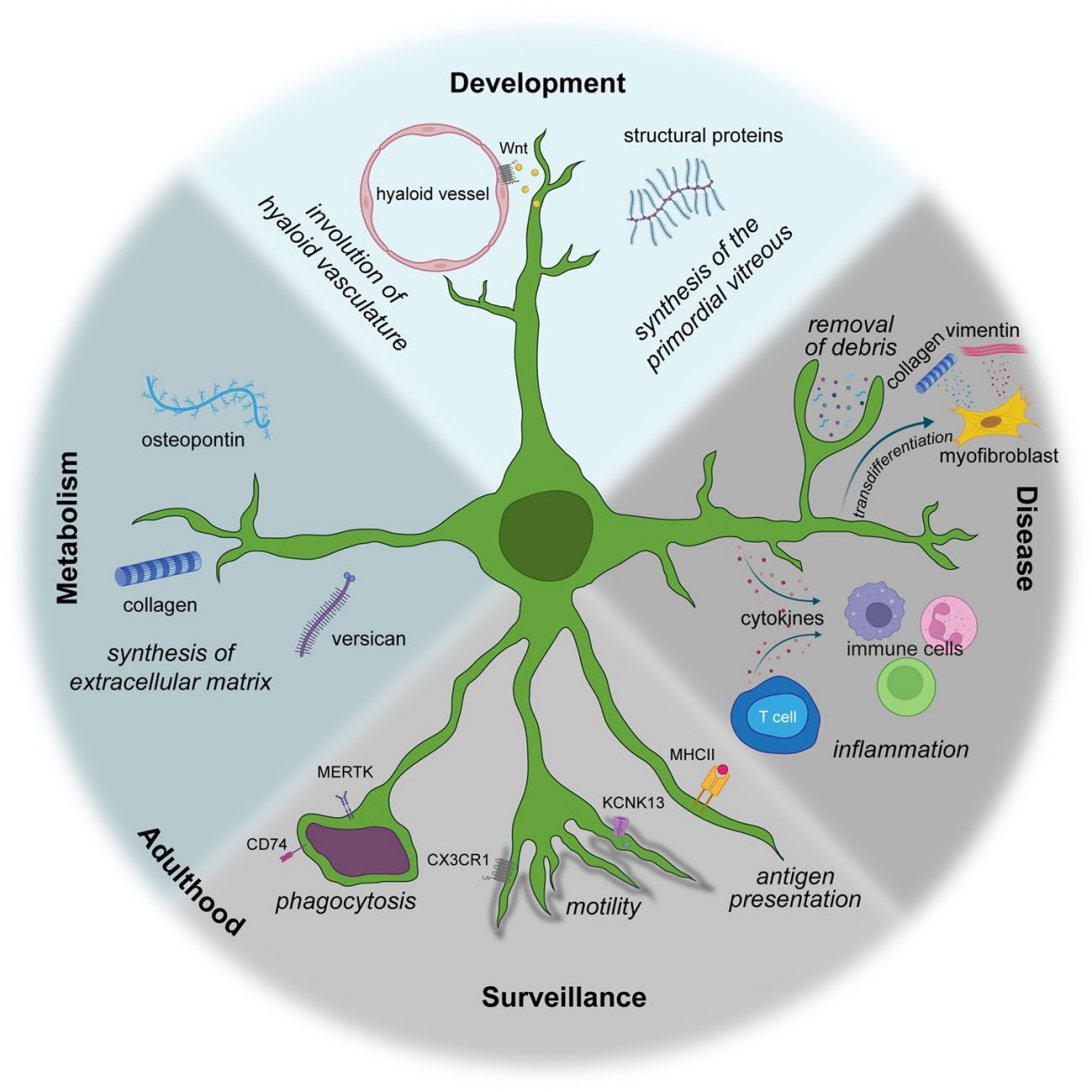
Functions of hyalocytes during development, adulthood, and disease. Hyalocytes exhibit a diversity of functions according to the developmental stage and health status: In the course of Wnt-mediated pre-programmed tissue-remodeling, hyalocytes play a role in the physiologic regression of the hyaloid vasculature during development of the eye, essential for a clear optical axis. Furthermore, hyalocytes might contribute hyaluronan and other structural molecules during formation of the secondary vitreous. Under steady-state postnatal conditions, hyalocytes play a role in vitreous metabolism and homeostasis by producing extracellular matrix components, such as secreted phosphoprotein 1 (SPP1, osteopontin), different collagen types, and versican. As part of their surveillance function, hyalocytes participate in phagocytosis, as evidenced by the expression of MERTK (MER proto-oncogene, tyrosine kinase) and CD74 (cluster of differentiation 74). Furthermore, hyalocytes are capable of scanning their environment by extending and retracting their projections, in line with the surface expression of important cell motility markers, such as KCNK13 (potassium channel, subfamily K, member 13) and CX3CR1 (CX3C chemokine receptor 1). In the course of inflammation, hyalocytes abundantly express MHCII (major histocompatibility complex class II) molecules, participate in removal of debris, and present antigens to T cells, which themselves contribute to the chemotaxis of other immune cells by cytokine expression. In proliferative vitreoretinal diseases, hyalocytes can transdifferentiate into α-SMA (alpha smooth muscle actin)-positive myofibroblasts, capable of producing collagen and vimentin, and thus contribute to the fibrotic nature of these conditions, as well as induce pathologic membrane contraction. Reproduced with permission from Boneva et al. [14]

Hyalocytes play various vital roles in the vitreous body of the developing and aging eye (Fig. 3). While our understanding of their function(s) is still evolving, several key roles have already been identified (for a detailed review, see [14]).

### Regression of fetal hyaloid vasculature

The mechanisms underlying the involution of the hyaloid vasculature are not yet fully understood, but several experimental studies provide insights into the role of hyalocytes in this process. Existing knowledge suggests an involvement of hyalocyte migration into the adventitia of primordial vitreous blood vessels and a destruction of endothelial cells adjacent to hyalocytes [16]. For this effect, the activation of Wnt signaling by *Wnt7b*, expressed in murine hyaloid macrophages, appears to initiate cell apoptosis in endothelial cells, ultimately resulting in the regression of hyaloid vessels [19]. This concept is supported by the observation of persistent hyaloid vasculature in mice deficient in *Ndp*, *Lrp5*, or *Fzd4*, which indicates a critical regulatory role for Wnt signaling [29]. Other factors implicated in hyaloid regression and modulation of hyalocyte function include von Hippel-Lindau protein, hypoxia-inducible factor 1 alpha, vascular endothelial growth factor, collagen XVIII, angiopoietin-2, and bone morphogenetic protein-4 [14, 30]. Furthermore, vitreous hyalocytes have been identified as the sole cell type expressing all four forms of transforming growth factor beta (TGF-β) in the eye [31]. The production of TGF-β by hyalocytes may contribute to apoptosis during the regression of hyaloid vasculature, as evidenced by the localization of TGF-β2 in the human vitreous during early developmental stages correlating with receding hyaloid vessels [32].

### Extracellular matrix synthesis

The adult human vitreous body consists of water (98%), structural proteins, mainly collagen and hyaluronan, and other ECM components like chondroitin sulfate [33, 34]. Historically, Schoeler, Virchow, and later Szirmai and Balazs assumed that hyalocytes contribute to vitreous anabolism, likely through hyaluronan production [34]. Already in 1960, Balazs noted an increased collagen concentration in vitreous of individuals aged 70–90 compared to younger groups, which was attributed to age-related vitreous gel volume decrease and a simultaneously stable collagen content [6]. Supporting this idea, the studies by Boneva et al. revealed an abundant expression of ECM components like COL5A1 and COL9A2 by hyalocytes of elderly individuals, suggesting that hyalocytes synthesize structural proteins throughout aging [10]. It is plausible that collagen synthesis by hyalocytes, rather than concentration increase due to volume decrease, contributes to the observed elevated collagen levels in the senescent vitreous, a notion supported by the presence of type II procollagen, a collagen precursor, in adult vitreous [33].

Unlike collagen, adult hyalocytes show relatively low expression of key factors responsible for hyaluronan synthesis, as observed in Boneva et al.’s 2020 study [10]. Interestingly, their research also highlighted increased RNA expression of versican and collagen type IX in aged hyalocytes. These proteins, in conjunction with hyaluronan, fibulin-1, and fibulin-2, play essential roles in maintaining the molecular structure and transparency of the vitreous body [35]. Changes in versican and minor glycosaminoglycans levels have been suggested as factors contributing to the liquefaction of vitreous with age, e.g., by Kamei et al. [36], indicating a potential role of hyalocytes in this process. Moreover, mutations affecting the splicing of chondroitin sulfate-bearing domains in versican have been associated with conditions characterized by excessive vitreous liquefaction, such as Wagner syndrome [37], further suggesting an involvement of hyalocytes in vitreoretinal dystrophies. In alignment with this notion, Boneva et al.’s research revealed that hyalocytes express high RNA levels of hyaluronidases, enzymes responsible for hyaluronan degradation, implicating their potential contribution to age-related liquefaction of the vitreous gel [10].

### Surveillance of the vitreoretinal interface

Already in the nineteenth century, Iwanoff (1865) and Potiechin (1878) postulated that hyalocytes are able to move in an amoeboid manner [3, 38]. Consequently, Schwalbe classified hyalocytes in the group of the wandering cells (‘Wanderzellen’), a term, which at that time referred to circulating and migrating macrophages and lymphocytes [4]. Recently, Castanos et al. documented human hyalocyte movement in vivo via OCT imaging [39] and subsequently employed AOSLO to image and map this movement (Fig. 2). They elegantly examined key characteristics of macrophage-like cells anterior to the inner limiting membrane (ILM) in healthy and diseased human eyes, which are likely to be hyalocytes. This was feasible in part because of the possibility to directly visualize these cells owing to their location anterior to the ILM. They found that human hyalocytes are distinctively distributed and have clear dynamic characteristics. As such, hyalocytes constantly explore their local environment by extending and retracting their projections within the timeframe of a few minutes, which is consistent with the hypotheses of Iwanoff and Potiechin proposed almost 150 years ago.

These current findings on cell motility are consistent with studies showing a high expression of the potassium channel *KCNK13* (potassium channel, subfamily K, member 13) and *CX3CR1* (CX3C chemokine receptor 1) in human hyalocytes [10] (Fig. 3), known to be important for microglia cell motility and immune surveillance [40]. In addition, hyalocytes were found to express membrane receptors that enable the cell to probe its environment, such as the adenosine A3 receptor, “sensing” ADP (adenosine diphosphate) released by, e.g., dying neurons and thus contributing to cell process extension [10]. Similar properties have been described for resting microglia in the brain and in the inner and outer plexiform layer of the retina [41]. Within minutes following acute injury, microglial processes converge toward the site of damage, and after hours to days, the reactive microglia retract their processes, form new motile protrusions, and transform into an amoeboid shape, thus migrating to the lesion. Chemotaxis of retinal microglia to sites of tissue damage, such as retinal neovascularization or retinal angiomatous proliferation [42], depends on activation of P2YR12 (purinergic receptor P2Y12) receptors on microglia that bind ATP (adenosine triphosphate) or ADP released from neural cells [43]. Since hyalocytes express *P2RY12* to a similar extent as human microglia [10], it is very likely that hyalocytes are capable of a similar reaction to tissue injury in disorders of the vitreoretinal interface, such as PDVR and PVR, where they migrate to sites of retinal inflammation or degeneration. This hypothesis would be in line with recent studies showing an increase of macrophage-like cells anterior to the ILM in patients with PDVR and PVR, which at least in part may be assembled by migrating and/or proliferating hyalocytes [12, 44], however, possibly also infiltrating monocytes from blood.

### Phagocytosis

Hyalocytes are capable of phagocytosis, the process of engulfing and removing cellular debris, foreign particles, and pathogens. Many decades ago, Hamburg proposed that hyalocytes play a role in clearing metabolic byproducts and maintaining the “hemato-ocular” barrier [45]. Their phagocytic capacity has been demonstrated both in vivo in rabbits and in vitro, confirming their affiliation with the mononuclear phagocyte system [46, 47]. Upon detecting injury or dying cells, hyalocytes are likely to migrate toward the harmful entities and phagocytose them, akin to microglia cells in the central nervous system. Recent evidence reveals that hyalocytes express factors involved in phagocytosis, such as *MERTK*, *CD74*, and *HLA-DRA*, which codes for MHCII molecules [10] (Fig. 3), suggesting their potential contribution to erythrophagocytosis and clearance of vitreous hemorrhage in conditions like PDVR or anomalous posterior vitreous detachment with vitreous hemorrhage.

However, other studies imply that hyalocytes may be activated following phagocytosis and may consequently have detrimental effects that exacerbate proliferative vitreoretinal diseases. As early as 1959, Hamburg proposed that hyalocytes contribute to intraocular fibrosis, based upon the assumed potential of hyalocytes to transform into fibroblasts and observations of increased hyalocyte numbers in patients with Coats disease [45]. Additionally, hyalocytes have been implicated in PVR formation not only by proliferating themselves, but by recruiting glial cells from the retina, retinal pigment epithelium (RPE), and monocytes from the circulation [13, 48]. Furthermore, recent evidence highlights transdifferentiation of hyalocytes into alpha-smooth muscle actin (α-SMA)-positive cells in the course of PDVR and PVR, underscoring their potential role in scar formation at the vitreoretinal interface [12, 10].

### Immunomodulation

Hyalocytes possess immunomodulatory properties and contribute to the regulation of immune responses within the vitreous body (for a detailed review, see [14]). As members of the mononuclear phagocyte system (previously called the reticulo-endothelial system), they are likely to interact with other immune cells, such as T cells and B cells, and thus influence immune signaling and cytokine production. Hyalocytes play a significant role in phagocyting harmful agents, such as foreign substances or microorganisms, which is crucial for antigen presentation and for triggering an immune response. By expressing MHCII-related genes like *HLA-DR*, hyalocytes have the potential to present antigens to CD4-positive T-lymphocytes (Fig. 3), similarly to retinal microglia [49]. However, the specific impact of hyalocytes on the adaptive immune response and their ability to either induce or suppress T cell function remain topics of ongoing discussion. Recent research suggests that the immunosuppressive properties of hyalocytes outweigh their pro-inflammatory activity. This balance helps to limit damage in the eye, thereby preventing irreversible neurodegeneration and maintaining optimal clarity along the optical axis. Hyalocytes further express cytokines, such as *SPP1* and *TNF* and many others, which are small signaling molecules that regulate immune responses and mediate cellular communication [10]. This cytokine production is very likely to influence the local microenvironment and immune cell behavior within the vitreous body in both health and disease [14].

### Immune privilege

Similar to the brain, the eye has long been recognized as an immune-privileged site [50]. This has historically been attributed to the absence of lymphatic drainage and the presence of blood-tissue barriers, leading to the concept of immunological ignorance, as proposed by Medawar [50]. Specifically, the notion of an immune deviation phenomenon emerged, defining an altered form of systemic immune response that occurs when antigens are introduced into privileged sites [51]. In the case of the eye, this response was initially termed anterior chamber-associated immune deviation (ACAID) [51], later expanding to vitreous cavity-associated immune deviation (VCAID) [52], and nowadays semantically refined as vitreous body-associated immune deviation (VBAID) to accurately reflect the nature of vitreous as an organ and not a space [14].

While ACAID involves bone marrow-derived antigen-presenting cells expressing F4/80 that convey ACAID-inducing signals to the spleen [53], it is assumed that VBAID is at least partly mediated by antigen-presenting hyalocytes [14]. This hypothesis is based on Sonoda’s experiments, which demonstrated delayed immune responses upon antigen injection into the vitreous body [52]. Mice previously inoculated with antigens showed reduced ear swelling when challenged with antigen-pulsed peritoneal exudate cells in the ear pinnae, as compared to controls. These findings suggest that immune deviation can also be induced in the vitreous body, similar to the anterior chamber, leading to systemic tolerance. Since F4/80-positive hyalocytes are the main cell population found in the vitreous body, they were proposed as the antigen-presenting cells responsible for mediating VBAID. It has been suggested, but not yet convincingly proven, that antigens introduced into the vitreous body are captured by hyalocytes and transported through the bloodstream to the spleen, where they stimulate natural killer T cells to produce immunosuppressive factors like IL-10 and TGF-β. This process may lead to the generation of antigen-specific regulatory T cells, shaping the adaptive immune response [52], which may reduce excessive inflammation within the eye and maintain immune privilege. However, hyalocytes may not only exert indirect immunosuppressive effects but may also directly contribute to an immunosuppressive environment within the vitreous body to preserve vitreous transparency and visual function. Recent evidence indicates that hyalocytes express various factors associated with immune privilege in the eye, including alpha-melanocyte-stimulating hormone (α-MSH), cluster of differentiation 86 (CD86), cluster of differentiation 46 (CD46), and TGF-β2, which can suppress inflammatory responses of T helper cells and induce regulatory T cells [10, 14]. However, further research is needed to fully understand the immunosuppressive mechanisms of hyalocytes in health and their potential pro-inflammatory properties in disease.

### Transdifferentiation of hyalocytes

Cell transdifferentiation represents the conversion of a differentiated cell type into another cell type. Due to the activation of new genes, cells thereby lose their original biochemical and morphological properties and transition into a new cell type. As far back as 1959, Hamburg suggested that hyalocytes might undergo a transformation into fibroblasts. He based this proposition on the observation of a higher concentration of hyalocytes in patients with Coats disease, specifically in cases of retrolental fibrosis [45]. This notion was later affirmed by in vitro studies showing that cultured hyalocytes overexpress α-SMA in response to TGF-β2. This was associated with a hypercontraction of collagen gels indicating a conversion of hyalocytes into myofibroblasts [54]. Recent evidence suggests that hyalocytes have the potential to transform also in vivo into α-SMA-positive myofibroblasts in proliferative vitreoretinal diseases. These myofibroblasts are capable of generating collagen and vimentin, thereby playing a role in the development of fibrosis associated with these conditions and contributing to the pathologic contraction of membranes in human vitreoretinopathies [10, 55].

In summary, hyalocytes exert important functions in the developing and aging vitreous body such as synthesis and degradation of vitreous components, phagocytosis of cellular debris and pathogens, and antigen presentation. Within the immune-privileged site of the eye, hyalocytes may have both indirect immunosuppressive effects through the induction of systemic tolerance upon antigen inoculation in the vitreous body, and direct control over intraocular inflammation through the expression of various immunomodulatory factors. Nevertheless, pernicious effects of hyalocytes in the setting of excessive scarring are likely and are the subjects of current research. It is important to note that our understanding of hyalocyte functions is still expanding, and ongoing research continues to uncover novel roles and nuances of their biology within the vitreous body.

## The roles of hyalocytes in vitreoretinal diseases

Hyalocytes have been suggested to play a significant role in autoimmune diseases, uveitis, and in proliferative vitreoretinal diseases characterized by hypercellular membranes, such as macular pucker, PDVR, and post-retinal detachment PVR. Additionally, they may be involved in paucicellular vitreo-maculopathies like macular holes and vitreo-macular traction syndrome. The common underlying features of these diseases are assumed to be anomalous posterior vitreous detachment and vitreoschisis (for a detailed review, see [13]). Before discussing in detail the role of hyalocytes in various vitreoretinal disease, it should be noted that the role of retinal microglia in vitreoretinal diseases is not addressed in this review (for a comprehensive review, see [56]) and that the distinct functions of retinal microglia from hyalocytes are still largely elusive. Preclinical evidence unequivocally illustrates the dynamic behavior of retinal microglia, which often leave their typical habitat in the plexiform layers to perform tasks such as phagocytosis of photoreceptors [57], or accumulate at the vitreoretinal interface in sites of retinal neovascularization [58, 59]. Transcriptomic analyses of human samples obtained from enucleated eyes have revealed a remarkable degree of similarity between isolated hyalocytes and retinal microglia, with approximately 97.8% of genes showing comparable expression levels in both cell populations. Despite this high degree of similarity, further analysis unveiled numerous genes that were significantly upregulated in hyalocytes as opposed to retinal microglia, which contribute to biological processes such as angiogenesis, chemotaxis, and leukocyte differentiation suggesting a distinct role for hyalocytes in specific vitreoretinal pathologies [26]. While further studies are needed to fully evaluate the different roles of microglia and hyalocytes in various vitreoretinal diseases, we focus below on the current evidence on hyalocytes in vitreoretinal diseases.

### Autoimmune diseases and uveitis

As innate immune cells, hyalocytes likely play a significant role in modulating inflammatory diseases of the posterior segment, including uveitis. Previous studies have postulated that hyalocytes may function as inhibitors of intraocular inflammation within the context of ocular immune privilege [10, 14]. Under normal conditions, it is assumed that the immunosuppressive properties of hyalocytes outweigh their pro-inflammatory antigen-presenting properties (see above) to maintain vitreous transparency. However, in uveitis, this balance appears to be upset, resulting in local inflammation and accumulation of vitreous cells, leading to clinical manifestations of uveitis, such as vitreous opacities, the visual phenomenon of floaters, and if severe, vision degrading myodesopsia [60]. Research using animal models such as endotoxin-induced murine uveitis and equine recurrent uveitis has demonstrated an increase in MHCII-positive phagocytes (most likely hyalocytes), particularly on the apical processes of the ciliary body adjacent to the anterior vitreous cortex [61, 62]. In vivo imaging studies in mice with uveitis have shown an accumulation and subsequent disappearance of CD68-positive and Cx3cr1-positive tissue-resident myeloid cells, including retinal microglia and hyalocytes in the course of disease [63]. However, it remains unclear whether the observed increase in myeloid cell numbers is due to recruitment of blood-derived macrophages or clonal expansion of hyalocytes. In vitro studies have shown that tumor necrosis factor-alpha (TNF-α), an established cytokine involved in uveitis and mainly expressed by macrophages, promotes hyalocyte proliferation, migration, and gel contraction [64]. Yet, the precise role of hyalocytes in uveitis is not well understood, and it can only be speculated whether the clinically commonly observed obstruction of fundus details is due to proliferating hyalocytes, infiltrating blood-derived immune cells, and/or protein exudation from inflamed blood vessels. The options for direct analysis of hyalocytes in human uveitis are limited, as primary vitrectomy is not commonly employed for treatment. Therefore, new diagnostic methods such as vitreous biopsy prior to intravitreal drug injections [65] and adaptive optics imaging approaches are needed to elucidate whether hyalocytes tend to suppress the inflammatory response and reduce tissue damage, or rather promote inflammation. Given the close relationship between vitreous hyalocytes and retinal microglia and their mutual roles in autoimmune and neurodegenerative disorders, it is tempting to speculate about the existence of different subpopulations of these cells and their respective functions in diseases like multiple sclerosis, which may present with intermediate uveitis in the eye [66].

### Age-related macular degeneration

Age-related macular degeneration (AMD) is a progressive chronic disease affecting the choroid, RPE, and neural retina. In its most aggressive form, known as neovascular AMD (nAMD), it is characterized by the infiltration of immune cells and myofibroblasts, and the accumulation of various extracellular matrix factors [67]. Previous research indicates a correlation between vitreo-macular adhesion and traction and an increased risk of choroidal neovascularization (CNV) in AMD [68], as well as a lower occurrence of CNV in patients with complete posterior vitreous detachment (PVD) or following vitrectomy [69–71] suggesting a potential role of vitreous and hyalocytes in the progression of this disease. Furthermore, eyes with exudative AMD and no PVD have a reduced responsiveness to anti-VEGF injections compared to those with PVD [72]. The mechanisms underlying these observations are poorly understood, but could involve a prevention of macular oxygenation and sequestration of pro-angiogenic cytokines by the attached posterior vitreous cortex, as well as pro-inflammatory effects of anomalous PVD with persistent vitreo-macular traction. On a cellular and molecular level, recent extensive studies on the composition of neovascular complexes in humans have shed light on the involvement of inflammatory cells, including microglia and blood-derived macrophages, in the formation of CNV [67]. Wieghofer and colleagues elegantly demonstrated that innate immune cells, such as retinal microglia and potentially vitreous hyalocytes, are the predominant immune cells at sites of experimental CNV and that monocyte-derived macrophages play a quantitatively lesser role in infiltration [25]. However, the role of innate immune cells, such as microglia and hyalocytes in AMD seems to be complex, as they most likely exert both beneficial and detrimental effects on disease progression. While they may be contributing to tissue damage and scarring through immune cell recruitment, they may also be promoting tissue repair and eliciting anti-inflammatory responses [73, 74]. However, the precise mechanisms by which the vitreous influences nAMD progression remain unclear and the specific role of hyalocytes in AMD has not been extensively studied. Yet, it is intriguing to speculate whether hyalocytes together with microglia might migrate to the neovascular complex and sustain an inflammatory stimulus that promotes neovascular development and/or persistence in AMD eyes with an attached posterior vitreous. Further research is needed to elucidate these mechanisms and the contribution of hyalocytes in AMD, both neovascular and dry.

### Anomalous posterior vitreous detachment (APVD) and vitreoschisis (VS)

**Fig. 4:**
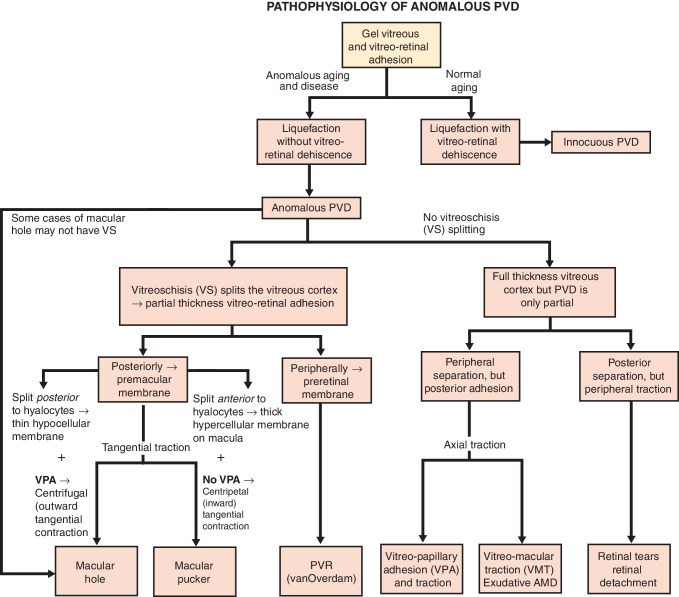
Anomalous posterior vitreous detachment (APVD). The various possible manifestations of APVD are demonstrated in this flow diagram. When gel liquefaction and weakening of vitreoretinal adhesion occur concurrently, the vitreous body separates away from the retina without sequelae. If the gel liquefies without concurrent vitreoretinal dehiscence, there can be various untoward consequences. If separation of vitreous from the retina is full-thickness but topographically incomplete, different forms of partial PVD occur (right side of diagram): Posterior separation with persistent peripheral vitreoretinal attachment can induce retinal breaks and detachments. Peripheral vitreoretinal separation with persistent full-thickness attachment of vitreous to the retina posteriorly can induce traction upon the macula, where it promotes neovascular age-related macular degeneration (AMD), and optic disc, where it contributes to neovascularization and vitreous hemorrhage in ischemic retinopathies. If during PVD the posterior vitreous cortex splits (vitreoschisis), there can be different effects depending on the level of the split (left side of diagram): Vitreoschisis anterior to the level of the hyalocytes (see Fig. 5) leaves a relatively thick cellular membrane attached to the macula and/or peripheral retina. In cases of retinal detachment, peripheral vitreoschisis leaves hyalocytes attached to the retina where they promote proliferative vitreo-retinopathy. Posteriorly, with separation from the optic disc (present in about 90% of cases) inward (centripetal) contraction of this premacular membrane induces macular pucker. If the split occurs at a level posterior to the hyalocytes (see Fig. 5), the remaining premacular membrane is relatively thin and hypocellular. Persistent vitreopapillary adhesion (VPA, present in 87.5% or more of cases) influences the vector of force in the tangential plane, resulting in outward (centrifugal) tangential traction (especially nasally) and inducing a macular hole. Some cases of macular hole may develop in the absence of vitreoschisis. Modified with permission from Sebag et al. [7]

In young individuals, the vitreous body is a transparent gel consisting primarily of water (98%) along with structural macromolecules (collagen and hyaluronan), and other important components of the extracellular matrix [34]. However, with aging, myopia, and diabetes, vitreous undergoes fibrous liquefaction and degeneration, causing destabilization of its structure. If there is a simultaneous weakening of the adhesion between the posterior vitreous and retina, the vitreous body can detach from the retina, resulting in an innocuous posterior vitreous detachment (PVD) [75, 76]. In cases where there is excessive fibrous liquefaction and degeneration within the vitreous body, coupled with insufficient weakening of vitreoretinal adhesion, an anomalous PVD can occur [76]. This can lead to various consequences depending on the location of the vitreoretinal separation and whether the outer vitreous layer (posterior vitreous cortex), which is lamellar, remains intact (Fig. 4). Splitting between the layers of the posterior vitreous cortex, referred to as vitreoschisis, is particularly important in the pathophysiology of proliferative vitreo-retinopathies [77–79].

Anterior displacement of the posterior vitreous inanomalous PVD and vitreoschisis most likely leads to altered vitreoretinal homeostasis, initiating a complex cascade of cellular responses. If the vitreoschisis split is relatively anterior, then many hyalocytes will be left attached to the retina. As a consequence, they will recruit immune cells from the bloodstream, glial cells from the retina and retinal pigment epithelial cells to the vitreoretinal interface, all of which can potentially cause and exacerbate proliferative vitreoretinal diseases. Although no direct evidence is yet available, it is very likely that hyalocytes act as sentinel of the vitreoretinal interface and mediate this cascade. Indirect evidence for this is the observation that hyalocytes express numerous signaling molecules that can initiate cell recruitment [10, 26]. However, it is important to note that not all patients with vitreoschisis develop proliferative vitreoretinal disease. This may be due to a relatively posterior split in the posterior vitreous cortex, leaving relatively few hyalocytes attached to the retina. Therefore, this hypothesized cascade of events appears to be highly individualized and dependent on factors such as the antero-posterior level and extent of cleavage, pre-existing vitreoretinal traction and breaks in the ILM, and possible disruption of the blood-retina barrier, although these remain so far unproven.

## Hypercellular tractional vitreo-retinopathies

Hyalocytes play a significant role in hypercellular, premacular membrane formation, contributing greatly to the development of macular pucker (MPK), proliferative vitreo-retinopathy, and proliferative diabetic vitreo-retinopathy. On the other hand, paucicellular premacular membranes contain primarily glial cells [80].

### Macular pucker

Macular pucker (MPK) was first described by Iwanoff in 1865 as a fibrocellular membrane with folds and striae in the inner retina [38], resulting in disturbances in the cytoarchitecture of the outer retina [81]. Although the term “epiretinal membrane” is often used to refer to this disease, it actually does not refer to the condition, but the membrane that causes MPK. Furthermore, the more accurate term for this membrane is “premacular membrane (PMM)” since this more precisely identifies the exact location of the membrane causing MPK. Previously labeled as “idiopathic,” it is now understood that MPK is caused by vitreous pathology. Early theories on the development of MPK focused solely on retinal factors and mostly disregarded the role of vitreous. Contemporary theories propose two main causes: breaks in the inner limiting membrane (ILM) leading to glial cell migration, and on the other hand anomalous PVD with vitreoschisis, which involves hyalocytes. PVD is present in 80–95% of MPK cases [82, 83], a significantly higher prevalence compared to the general population over the age of 50 [84]. The *retinal break/glial cell theory* suggests that microbreaks in the ILM due to PVD create pathways for glial cells to migrate and proliferate along the retinal surface [85]. These assumed retinal breaks have, however, so far not been documented on histologic examination. The *hyalocyte theory* directly implicates a role of the vitreous, via anomalous PVD with vitreoschisis. Spectral-domain OCT studies have detected vitreoschisis in 42% of MPK eyes [48], with a higher prevalence likely to be reported in future studies using superior imaging technologies, such as swept-source OCT, since during surgery vitreoschisis was found in 80% of MPK eyes [86].

**Fig. 5:**
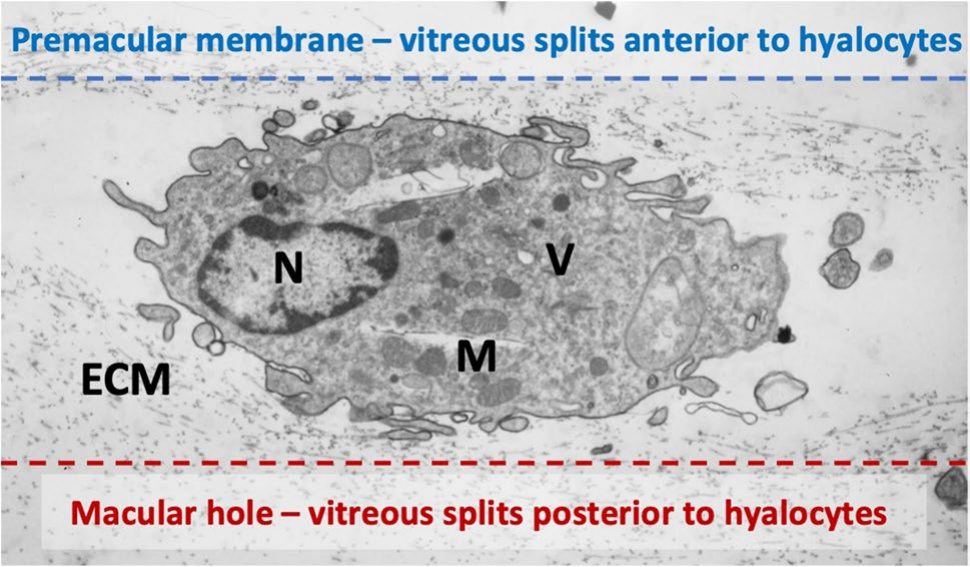
Proposed hypothesis of the role of hyalocytes in premacular membrane and macular hole formation. Ultrastructure of vitreoschisis. Human hyalocyte, as imaged by transmission electron microscopy, embedded in the collagen meshwork of the posterior vitreous cortex (original magnification × 11,670). Vitreoschsis anterior to the level of hyalocytes upon anomalous posterior vitreous detachment leaves a relatively thick, hypercellular membrane attached to the macula (dotted blue line). Inward (centripetal) contraction of this membrane induces macular pucker. If the split occurs at a level posterior to the hyalocytes, the remaining premacular membrane is relatively thin and hypocellular (dotted red line). Outward (centrifugal) tangential traction can induce a macular hole. Modified with permission from Sebag et al. [27]

The level at which vitreoschisis occurs influences the pathology. If the posterior vitreous cortex splits more anteriorly, a greater number of hyalocytes will remain adherent to the macula within the premacular membrane, initiating the process of MPK formation by recruiting monocytes and glial cells (see above). Hyalocytes embedded in this vitreous layer have been observed within premacular membranes excised from MPK patients [87] (Fig. 5). Additionally, hyalocytes can transform into myofibroblasts, causing tangential membrane contraction and exerting centripetal (inward toward the fovea) forces on the retina, resulting in the characteristic irregular retinal contour seen in MPK.

The aforementioned pathological mechanism may also underlie lamellar hole-associated epiretinal proliferation (LHEP), as cellular ultrastructural analysis of surgically excised tissue revealed mainly fibroblasts and hyalocytes in LHEP [88]. Although there are other theories for the development of LHEP postulating a Müller cell-driven process in response to factors such as degenerative changes in the retina [89], a role for hyalocytes in the development of LHEP, whether primary or secondary reactive, is highly likely.

Currently, vitrectomy with membrane peeling is the established treatment for MPK, and visual outcomes depend on factors such as the presence of multiple areas of retinal contraction, macular edema, alterations in the photoreceptor layer at time of surgery, and the integrity of the inner segment/outer segment junction [77, 90]. Whether the prophylactic induction of a complete PVD or the molecular modulation of hyalocytes can be a viable therapeutic approach for the prevention of MPK in the future needs to be investigated in more detail.

### Proliferative vitreo-retinopathy

Proliferative vitreo-retinopathy (PVR) is a fibroproliferative disorder hallmarked by the formation of contractile, fibrocellular membranes in, on, and/or beneath the retina, that can arise following rhegmatogenous retinal detachment (RRD), surgical procedures, or trauma. Surgical failure after RRD repair occurs in 10–15% of cases and is often attributed to the development of PVR in the peripheral fundus [91]. PVR is characterized by three stages: inflammation, cell proliferation, and extracellular matrix (ECM) remodeling. Early stages of the disease exhibit marked hypercellularity, partly due to an anterior splitting of the vitreous cortex during anomalous PVD with vitreoschisis. The primary cell types involved in PVR include hyalocytes, retinal microglia, astrocytes, retinal Müller cells, RPE cells, and myofibroblasts [55, 92]. Since they reside in the posterior vitreous cortex, hyalocytes have been suggested to be particularly important in initiating and promoting PVR by modulating immune and inflammatory processes, triggering fibrosis, and undergoing transdifferentiation into myofibroblasts, which eventually leads to the formation of tractional fibrocellular membranes [13, 80, 93]. Later in the pathophysiology of PVR, other myeloid cells such as retinal microglia and infiltrating blood-derived monocytes may also play a role. The final fibrotic stage is characterized by membrane maturation and tangential contraction that is centripetal, thus causing underlying retinal folds.

**Fig. 6:**
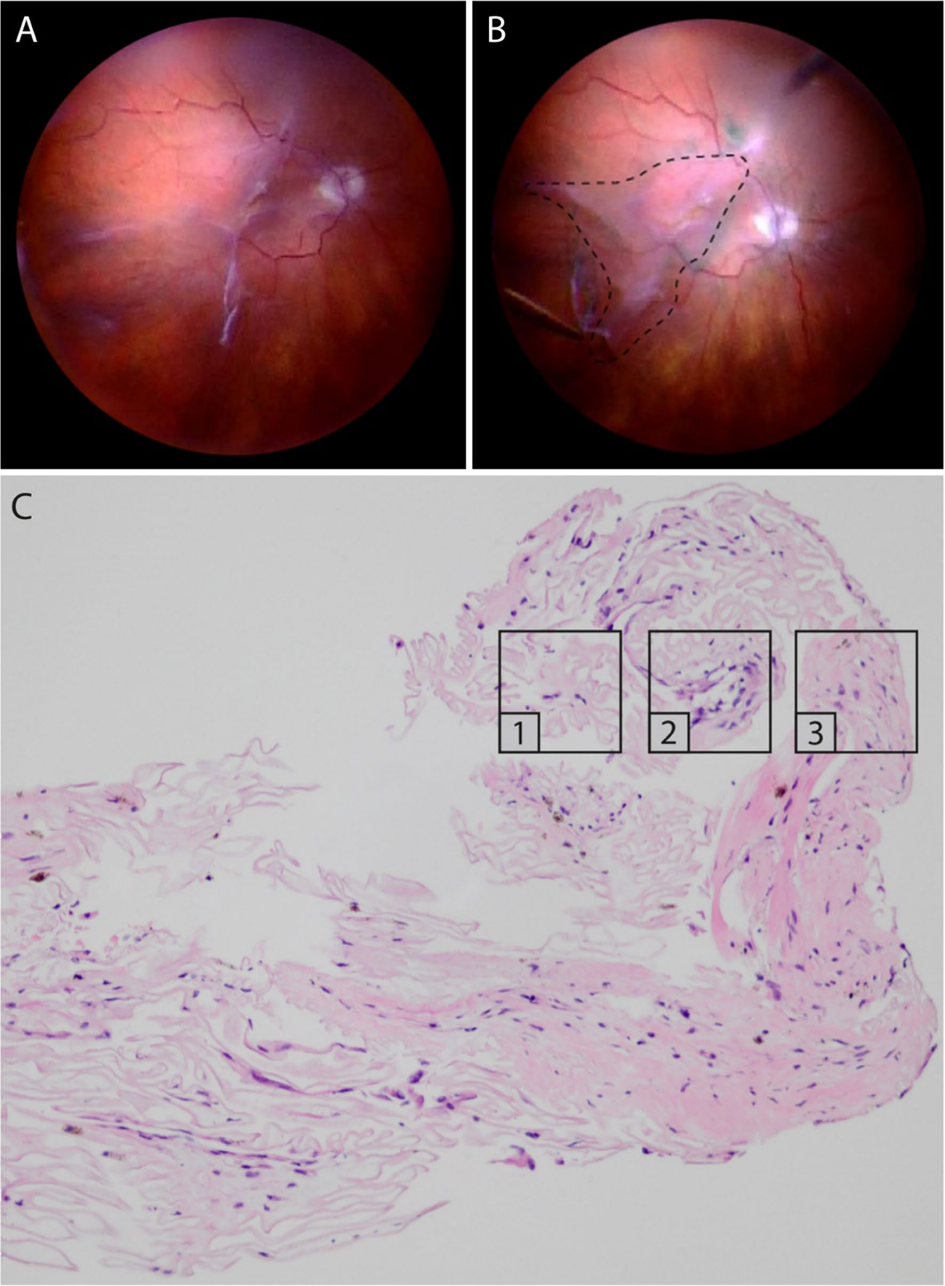
Proliferative vitreo-retinopathy (PVR). Intraoperative imaging before (**A**) and after (**B**) peeling of a PVR membrane, extending from the superior arcade to the inferotemporal periphery. The dashed line indicates the area from where the membrane was excised. **C** Light microscopy of the PVR membrane stained with hematoxylin and eosin. Different continuous membrane areas can be distinguished, representing different stages of PVR: paucicellular, lamellar collagen-rich areas with hyalocytes, suggestive of VCR (1); areas with increased cellular infiltration by glial cells and pigmented cells (possibly hyalocytes with engulfed pigment or RPE cells) (2); more fibrotic areas with low cellularity and myofibroblasts (3). Courtesy of and used with permission of Dr. Koen van Overdam

Several studies and extensive surgical experience underscore the crucial role of vitreous in PVR, since the vitreous cortex is a scaffold for fibrocellular proliferation and an active participant in ECM remodeling. Within this structure, there exists an abundance of profibrotic and inflammatory mediators capable of inducing ECM contraction via hyalocytes [93, 94]. In similar fashion to the vitreo-macular interface, the lamellar structure of the peripheral vitreous cortex predisposes to vitreoschisis (VS), an important initiating event in PVR [34, 76]. Resulting residual vitreous cortical remnants (VCR), serve as scaffolds for PVR development, particularly if VS causes splitting of the posterior vitreous cortex anteriorly, leaving many hyalocytes within the VCR. Histopathological analysis of PVR membranes supports the association between VCR and PVR, as both native collagen and newly formed ECM components are detected within [95]. Within PVR membranes, different areas exhibit distinct cell and ECM characteristics. Areas characterized by low cellularity with hyalocytes and abundant collagen, likely correspond to VCRs resulting from VS [96] (Fig. 6). Hyalocytes play a critical role by recruiting circulating monocytes (see above), secreting profibrotic cytokines, inducing ECM synthesis, promoting myofibroblast differentiation, and driving ECM contraction [12, 93, 97]. Additionally, imaging mass cytometry demonstrated the presence of IBA1-positive myeloid cells (possibly vitreous hyalocytes and/or retinal microglia) co-expressing α-SMA in surgically excised preretinal PVR membranes, suggesting a transdifferentiation of myeloid cells into myofibroblasts as a common feature in PVR formation [12].

### Proliferative diabetic vitreo-retinopathy

Diabetic retinopathy is the primary cause of severe vision loss in working-age adults and poses a significant medical and socio-economic challenge due to its increasing prevalence [98]. Proliferative diabetic vitreo-retinopathy (PDVR), an advanced stage of the disease, is characterized by poorly perfused retina and hypoxia triggering the release of pro-angiogenic growth factors, leading to retinal and optic disc neovascularization [99]. While intravitreal anti-vascular endothelial growth factor (VEGF) therapy effectively inhibits VEGF and is commonly used to treat diabetic macular edema and PDVR, some patients exhibit only a moderate or poor response to this approach [100]. In these patients, contractile vascular membranes may form at the vitreoretinal and vitreo-papillary interface. Clinical OCT studies have recently revealed clustering of plumper cells (most likely hyalocytes) around neovascularization sites and altered VRI cell morphology in patients with PDVR [39, 44]. Immunohistochemical studies reveal that hyalocytes in these eyes are situated directly on the retinal surface or within the anterior-most vitreous collagen fibrils. Immune cell markers, such as CD45, CD64, and IBA1, have been detected in these cells [101, 102]. In a recent study, Boneva et al., found that retinal neovascular complexes in PDVR contain myeloid cells, partly double positive for IBA-1 and α-SMA, indicating their likely identity as hyalocytes based on their proximity to preretinal PDVR membranes, but also their potential to transdifferentiate in myofibroblasts [10]. *TGF-β*, a cytokine known to induce α-SMA expression in myeloid cells and promote hyalocyte contraction [103], was significantly upregulated in human retinal neovascularization on the transcriptional level [10]. These findings suggest that TGF-β-mediated myofibroblast transdifferentiation of hyalocytes plays a crucial role in the formation of contractile fibrovascular membranes in advanced PDVR.

### Paucicellular tractional vitreo-maculopathies

The term paucicellular tractional vitreo-maculopathies encompasses a group of macular pathologies characterized by vitreo-macular adhesion, and formation of fibrocellular membranes with relatively few cells. This group includes vitreo-macular traction syndrome (VMTS), macular holes, and myopic foveoschisis [13, 104]. In VMTS, age-related fibrous degeneration of the vitreous body along with simultaneous persistent adhesion of the posterior vitreous cortex to the ILM causes traction in an axial (anterior–posterior) direction. Cell clusters on the ILM mark the initial stage of pathologic membrane formation in VMTS [102, 105]. In studies using correlative light and electron microscopy, hyalocyte activation has been implicated in vitreo-macular traction development despite low cellularity [106, 107]. Additionally, transdifferentiation of hyalocytes into myofibroblasts was suggested to contribute to the composition of fibrocellular membranes [106, 108]. A further understanding of the cellular dynamics and molecular processes involved in paucicellular tractional vitreo-maculopathies and elucidation of the role of hyalocytes in their pathogenesis would provide insight into potential therapeutic strategies.

## Therapeutic and preventive approaches for proliferative vitreo-retinopathies

Despite increased understanding of the crucial roles played by various cells in proliferative vitreo-retinopathies, targeted therapeutic strategies for preventing conditions such as macular pucker, PVR, vitreo-macular traction syndrome, and macular hole are currently lacking [109]. However, advanced surgical techniques with chromodissection [110] represent a potential solution for facilitating the complex and challenging maneuver of membrane dissection at the vitreoretinal interface by providing better visualization and minimizing surgical trauma to the underlying retinal nerve fibers. This approach aims to thoroughly excise the peripheral vitreous that remains attached to the ILM due to vitreoschisis, thereby eliminating any VCR containing hyalocytes and their stimulatory effects [79, 96, 111]. By removing the scaffold for cell migration, proliferation, and pathologic extracellular matrix synthesis and contraction, during the initial repair of retinal detachment, chromodissection has demonstrated improved surgical outcomes by reducing the risk of recurrent retinal detachment and premacular proliferation with PVR as well as macular pucker [79, 112, 113]. For effective vitrectomy in primary and recurrent retinal detachment cases, van Overdam and colleagues recommend the complete removal of vitreous using targeted triamcinolone acetonide-assisted visualization and chromodissection techniques, including vitreous shaving, indentation at the vitreous base, and detection and removal of VCR over the macula and peripheral retinal surface, particularly when other PVR risk factors are present [96, 111]. Although van Overdam and other independent research groups found that this technique reduced PVR [113–115], further studies by different groups and surgeons are needed to reliably assess the impact on PVR and RD recurrence. Moreover, it is important to note that the detection and removal of vitreous cortex remnants pose considerable challenges, often requiring substantial time and carrying the inherent risk of iatrogenic damage to retinal tissue. Consequently, there is a pressing need for advancements in both the detection and removal techniques of VCR, possibly employing adjuvant therapies such as pharmacologic vitreolysis [116]. Additionally, comprehensive studies are warranted to delineate patient demographics that would derive maximal benefit from VCR removal, as well as to ascertain the optimal extent of VCR removal necessary for clinical efficacy. Finally, the distribution of hyalocytes following vitrectomy is currently elusive. Theoretically, complete vitreous removal should eliminate all hyalocytes from the eye. Nonetheless, residual vitreous may persist despite meticulous vitrectomy, particularly in the ciliary body region, renowned for its hyalocyte abundance and firm vitreoretinal adhesion. The potential for hyalocytes to re-populate the retinal surface, and the possible mechanisms underlying this process, which may involve clonal expansion or migration of macrophages from the ciliary body and iris, akin to observations in murine retinal microglia following pharmaceutical depletion [117], remain an enigma awaiting future investigation.

## Concluding remarks

Despite being the largest structure in the human eye, the vitreous body remains one of the least understood of all ocular tissues regarding its roles in both health and disease. Consequently, there is limited knowledge about various aspects of vitreous physiology and how it maintains a stable microenvironment within the eye. One crucial function of vitreous is to ensure transparency in the optical axis, allowing unimpeded light transmission to the retina. The rigorous organization of vitreous macromolecules minimizes light scattering, although this changes with age and certain diseases. Transparency also necessitates a relatively cell-free environment, yet the presence of cells within the vitreous body has been a subject of controversy since their discovery almost two centuries ago. Based on the evidence presented, it can be reasonably concluded that hyalocytes are a distinct population of cells residing in the vitreous body, separate from retinal microglia, despite their many similarities. While hyalocytes are distributed throughout the vitreous body, those located within the posterior vitreous cortex anterior to the ILM of the retina may play a crucial role. These hyalocytes act as sentinel cells, guarding against any harmful conditions that threaten the posterior segment and, indeed, the entire eye. Hyalocytes in the anterior vitreous and at the vitreous base may serve a similar function in relation to the ciliary body. The reactions of posterior hyalocytes to trauma, infection, aging, neurodegenerative disorders, and systemic diseases likely contribute to the pathophysiology of various posterior segment eye diseases.

As elucidated in this review, hyalocytes have significant involvement in proliferative vitreoretinal pathologies, including uveitis, both hypercellular and paucicellular vitreo-retinopathies, and possibly AMD. Residing in the posterior vitreous cortex, hyalocytes are early responders and likely play a significant role in disease progression by promoting cell migration, proliferation, transdifferentiation, and exerting contractile effects on the retina. Advances in visualizing hyalocytes in diseased eyes in vivo will improve our understanding of their contribution to pathogenesis. A deeper understanding of hyalocytes’ role(s) in early-stage proliferative disorders at the vitreoretinal interface could lead to novel treatment strategies, such as targeted therapies directed at these cells, thereby mitigating disease progression. Strategies aimed at impeding anomalous PVD and vitreoschisis could mitigate the effects of hyalocytes in proliferative vitreo-retinopathies.

To date, knowledge of vitreous anatomy, anomalous posterior vitreous detachment, vitreoschisis, and the role of hyalocytes has already led to the emergence and implementation of preventive strategies via meticulous surgical removal of peripheral vitreous and hyalocytes in avoiding PVR. Successfully eliminating the involvement of vitreous and hyalocytes in this disease serves as a model for other disease prevention. This could also be done by averting PVD. Alternatively, inducing a harmless PVD could prevent proliferative vitreoretinal diseases by eliminating hyalocyte involvement in pathogenesis. Exploring pharmacologic vitreolysis to induce prophylactic PVD could be a valuable avenue for future research and development, aiming to achieve similar benefits without the need for a surgical intervention [9, 116]. Considering the eye as a window to the body, the interaction between the eye and the central nervous system is particularly intriguing. Therefore, investigating hyalocytes in the posterior vitreous and their interaction with the neural retina could also provide insights into neurodegenerative disorders of the central nervous system.
